# Gut microbiome, T cell subsets, and cytokine analysis identify differential biomarkers in tuberculosis

**DOI:** 10.3389/fimmu.2024.1323723

**Published:** 2024-04-08

**Authors:** Yinghui Chai, Xin Liu, Guangliang Bai, Nannan Zhou, Danfeng Liu, Xiaomeng Zhang, Min Li, Kang Li, Hong Lei

**Affiliations:** ^1^ Department of Clinical Laboratory, the 8th Medical Center of People's Liberation Army (PLA) General Hospital, Beijing, China; ^2^ First Clinical Medical College, Hebei North University, Zhangjiakou, China

**Keywords:** gut microbiome, T cell subsets, cytokines, biomarkers, tuberculosis

## Abstract

**Introduction:**

The gut microbiota, T cell subsets, and cytokines participate in tuberculosis (TB) pathogenesis. To date, the mechanisms by which these factors interactively promote TB development at different time points remain largely unclear. In the context of this study, We looked into the microorganisms in the digestive tract, T cell types, and cytokines related to tuberculosis.

**Methods:**

According to QIIME2, we analyzed 16SrDNA sequencing of the gut microbiome on the Illumina MiSeq. Enzyme-linked immunosorbent assay was used to measure the concentrations of cytokines.

**Results:**

We showed the presence of 26 identifiable differential microbiomes in the gut and 44 metabolic pathways between healthy controls and the different time points in the development of TB in patients. Five bacterial genera (*Bacteroides*, *Bifidobacterium*, *Faecalibacterium*, *Collinsella*, and *Clostridium*) were most closely associated with CD4/CD8, whereas three bacterial taxa (*Faecalibacterium*, *Collinsella*, and *Clostridium*) were most closely associated with CD4. Three bacterial taxa (*Faecalibacterium*, *Ruminococcus*, and *Dorea*) were most closely associated with IL-4. *Ruminococcus* was most closely associated with IL-2 and IL-10.

**Conclusion:**

Diverse microorganisms, subsets of T cells, and cytokines, exhibiting varying relative abundances and structural compositions, were observed in both healthy controls and patients throughout distinct phases of tuberculosis. Gaining insight into the function of the gut microbiome, T cell subsets, and cytokines may help modulate therapeutic strategies for TB.

## 1 Introduction

Tuberculosis (TB) is a grave worldwide public health issue that poses a grave threat to human health (1). In 2022, the World Health Organization published the Global Tuberculosis Report, which showed that TB continues to be the contagious illness responsible for the highest number of fatalities globally. In 2021, China had approximately 780,000 new cases of TB, ranking second in the world, and is one of the 30 nations across the globe with a significant tuberculosis prevalence (2). *Mycobacterium tuberculosis* (MTB) can invade various organs of the human body; however, 80% of infection is through the respiratory tract, primarily targeting the lungs, causing TB (3). Statistics show that as many as 2 million people die of TB globally every year, and approximately 1 million new patients are diagnosed with TB annually in China (4).

Gut microbes have an important influence on the host’s immune response, and they can influence human health and disease course by regulating effector T cells in the gut. Studies have shown that certain gut microbes can promote the differentiation of specific T-cell subpopulations in the gut (5). *Bacteroides* and *Clostridium* can induce the differentiation of Treg cells (6). *Bordetella pertussis* can promote the maturation of Th1 cells in the gut (7). *Segmented Filamentous Bacteria*, *Citrobacter Rodentium*, and *E.coli* can induce the differentiation of Th17 cells in the mouse intestine (8). The infiltration of Th1 cells and Th17 cells in the lung is considered to be an important contributor to the immunopathogenesis of pulmonary tuberculosis (9). These cells regulate and activate other immune cells by producing a series of cytokines, such as interferon γ(IFN-γ), tumor necrosis factor-α (TNF-α), interleukin-17 (IL-17), etc. Such as macrophages and natural killer cells, together involved in the antagonism of mycobacterium infection.

Since the development of metagenomic technology, It has been extensively utilized in the examination of gut bacteria, and the understanding of the human intestinal flora has increased considerably (10). There is a strong correlation between TB and a variety of factors that can affect a person’s immunity, genetics, and the environment (11). However, recent studies have indicated a potential link between an imbalance of the intestinal microbiome and the emergence of tuberculosis. The relation between gut microbiota and inflammatory factors in TB patients is less analyzed.

We compared the gut microbiota profiles of TB patients with those of healthy individuals. The metabolic pathways of certain microbial genera in the intestines have been linked to tuberculosis. Our objective was to ascertain the connections between the microbiome and TB in order to facilitate the development of novel therapeutic alternatives for crucial medical conditions that impact the outcomes of disease treatment.

## 2 Materials and methods

### 2.1 Sample collection

We conducted metagenome sequencing on 90 fecal samples obtained from 30 individuals in the jk group, 30 patients with initial TB in the cz group, and 30 patients with recurrent TB in the fz group. No respiratory illnesses were present in any of the controls, and there was no record of any contact with individuals suffering from active TB. No one of the registered participants had been given probiotics, prebiotics, or antibiotics a month before being admitted to the hospital. The initial TB group was those who had never used antituberculosis drugs or were treated with antituberculosis drugs for the first time for less than 1 month; The Recurrent TB refers to patients with tuberculosis who have been treated with anti-tuberculosis therapy for more than 1 month or who have failed initial treatment and relapsed. All participants’ basic demographic and clinical data were gathered during the initial hospital assessment. The storage of fresh stool samples took place at a temperature of -80°C. Medical Ethics Committee, Eighth Medical Center, PLA General Hospital committee that approved this study.

Following the conclusion of treatment, The serum was gathered and spun in a centrifuge at 13,000 revolutions per minute for 10 minutes at 4°C. The biochemical indicators were detected after storing the supernatant at -80°C. Enzyme-linked immunosorbent assay was used to measure the concentrations of cytokines (NK, IL-2, IL-4, IL-6, IL-1β, IL-10, IL-17A, IL-21, IL-12p70, IFN-γ, and TNF) in the serum, following the manufacturer’s instructions with commercial ELISA kits (CUSABIO, China).

Whole blood samples were collected from the participants using venipuncture in a consecutive manner. A CyFlow Counter (Beckman Coulter FC_500, USA) was used to enumerate CD3+, CD4+, CD8+, and T cells, followed by data analysis using the CXP analysis software.

### 2.2 DNA extraction and PCR amplification

The E.Z.N.A.^®^ Stool DNA Kit (D4015, Omega, Inc., USA) was used to extract microbial DNA from the fecal samples according to the manufacturer’s guidelines. PCR was used to amplify the V3–V4 region of the bacterial 16S rRNA gene (95°C for 3 min followed by 25 cycles of 95°C for 30 s, 55°C for 30 s, and 72°C for 30 s, followed by a final extension at 72°C for 5 min) using the primers 338F (5’-ACTCCTACGGGAGGCAGCAG-3’) and 806R (5’-GGACTACHVGGGTWTCTAAT-3’), wherein an eight-base sequence represents a barcode exclusive to each sample. A triplicate 20 μL mixture was used for PCR reactions, comprising 4 μL of 5 × FastPfu Buffer, 2 μL of 2.5 μM dNTPs, 0.8 μL of each primer (5 μM), 0.4 μL of FastPfu Polymerase, and 10 ng of template DNA.

### 2.3 Sequencing data from Illumina MiSeq was sequenced and processed

Equimolar amounts of the purified amplicons were pooled, and then subjected to paired-end sequencing on an Illumina MiSeq PE300 platform (Illumina, San Diego, CA, USA) following the standard protocols established by Majorbio Bio-Pharm Technology Co., Ltd. (Shanghai, China).

The FASTQ files were imported in a format compatible with the QIIME2 (https://docs.qiime2.org/2019.1/) system using the QIIME tools import program. A sequence of demultiplexed sequences from each sample was quality filtered, trimmed, and de-noised, and then the QIIME2 dada2 plugin used to detect and eliminate chimeric sequences, resulting in the feature table of the amplicon sequence variant (ASV) (12). A taxonomy table was generated by aligning ASV sequences to a pre-trained SLVA database using the QIIME2 feature-classifier plugin, which was trimmed to the V3–V4 region bound by a 338 F/806 R primer pair (13). Various techniques, such as ANCOM (Analysis of Composition of Microbiomes), analysis of variance, Kruskal–Wallis, linear discriminant analysis effect size (LEfSe), and DEseq2, were utilized to distinguish bacteria with varying levels of abundance between the samples and groups (14).

### 2.4 Bioinformatics analysis

Alpha-diversity, which includes the richness and diversity of bacteria in the samples, was measured using the Chao1, ACE (abundance-based coverage estimators), Sobs, Shannon, and Invsimpson indices. The number of genera and their relative abundance in the gut was determined by measuring the diversity of microorganisms. The beta diversity of the gut microbiota was estimated using weighted UniFrac metric principal coordinate analysis (PCoA) based on the biological evolution information of the sequences from each sample, revealing differences in the gut microbiota community between groups. The “vegan” package was utilized to compute alpha diversity (15). The R package “encodes” was used to determine the Bray–Curtis dissimilarity between various sample types (16). Using the Galaxy Platform (https://huttenhower.sph.harvard.edu/galaxy/), we employed LEfSe to distinguish the cluster of representative bacteria (17). Continuous variables were compared using the Wilcoxon rank-sum test.

### 2.5 Classifier

We employed the random forest model, the most reliable ensemble machine learning technique for classification and regression, to construct the classifier with the relative abundance of bacterial taxa and the default parameter R algorithm ‘RandomForest’,’ ntree=1,000, based on the default metric of p/3 where p is the number of taxa of a class, in order to maximize the contribution of taxa to the gut microbiome (18).

### 2.6 Biomarker identification

Using the “glm” function of R software, stepwise logistic regression models were constructed to differentiate between paired groups. Biomarker identification was performed using a stepwise selection algorithm with the package MASS (19) in R. All significantly altered bacterial species were included in the models as potential biomarkers. The Akaike Information Criteria stepwise model selection algorithm was then used to identify the final biomarkers, using the R function “STEPAIC” from the MASS package. The R package caret was used to perform a 10-fold cross-validation of all identified biomarkers through random forest verification (20). Receiver operating characteristic (ROC) analysis was conducted to illustrate the performance of the classification models using the R package pROC (21). Interactions among disease-associated microbiomes were estimated using Spearman’s rank correlation. All heatmaps were drawn in R software using the “heatmap” package.

### 2.7 Network analysis

The co-occurrence network analysis encompassed the computation of bacterial correlations in both healthy control samples and TB patients, ascertained through the utilization of SparCC with 100 bootstraps to estimate the p-value, based on the relative abundance of each genus (22). We upheld the correlation values with a p-value below 0.05. Gephi was utilized to observe the relationship between stool samples from healthy controls and TB patients in terms of microbial community. The proximity and eigenvectors of the nodes were computed to assess the centrality of each node within the network.

## 3 Results

### 3.1 Characteristics of all participants

Ninety participants, including initial TB patients (cz, n=30), retreated patients (fz, n=30), and healthy controls (jk, n=30), were enrolled in this study. The clinical demographics of the study cohort are shown in **Table 1**. The participants in the three groups were paired based on their age, gender, and clinical symptoms. The comprehensive attributes of the registered participants are furnished. The white blood cell counts in the cz and fz group were higher than those in the healthy controls group, and percentage of lymphocytes in the healthy controls group had a significantly lower than cz and fz group (p < 0.05). The cz and fz group exhibited a notable decrease in hemoglobin levels compared to the control group (p < 0.05). No noteworthy distinctions were observed among the other indicators (p > 0.05) (**Table 1**).

**Table 1:** Comparison of demographic and clinical characteristics between the primary treatment, retreatment, and control groups.

	Jk (n = 30)	Cz (n = 30)	Fz (n = 30)	*H/F/Z*	*p*
Sex (m/f) Age WBC(10^9^/L) eosinophils (%) lymphocytes (%) neutrophils (%) ESR (mm/H) HGB (g/L) A/G AST (U/L)	15/15 39.36 ± 7.91 5.00 (4.37–5.65) 1.89 (1.32–2.60) 31.75 ± 4.88 59.30 ± 5.19 - 142.55 ± 13.72 1.44 ± 0.21 17.85 (12.05–22.58)	18/12 37.32 ± 14.59 6.49 (5.05–6.91) 1.60 (1.28–4.48) 25.17 ± 8.80 65.01 ± 9.91 10.00 (2.75–23.25) 136.86 ± 15.07 1.35 ± 0.29 16.50 (13.60–19.80)	14/16 39.9 ± 13.70 6.00 (4.93–7.39) 1.75 (1.02–3.63) 26.47 ± 11.60 63.87 ± 13.36 19.00 (5.25–25.00) 121.05 ± 21.35 1.39 ± 0.32 17.05 (14.20– 22.60)	- 2.02 7.97 0.31 3.46 2.02 -1.19 9.02 0.54 0.20	- 0.77 0.02 0.86 0.04 0.14 0.24 <0.01 0.59 0.90

### 3.2 Estimation of sequencing depth

16S rDNA sequencing of 90 samples was performed using the MiSeq sequencing platform. Following the process of quality control filtering, each sample yielded an average of 79,188 valid data points. The sequences were grouped together into 11,940 ASVs, all of which were identical. A total of 17,867 ASVs (74.99%) were classified according to their genus.

**Figure 1:**
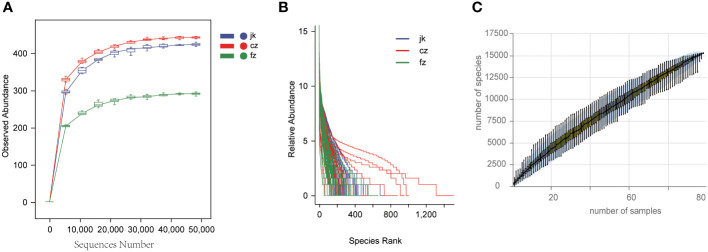
Estimation of sample depth in the cz, fz, and jk groups. **(A)** Rarefaction curves. **(B)** Rank Abundance curves. **(C)** Species Accumulation Boxplots. cz, initial TB patient; fz, recurrent TB; jk, healthy control.

The rarefaction curves for each sample group advanced to the platform stage, suggesting a substantial amount of sequencing data (**Figure 1A**). The rank abundance curves depicted a high level of richness and uniformity in every sample (**Figure 1B**). As the sample size increased, the species accumulation boxplot demonstrated a steady rise in species diversity, with the curves becoming less steep when the sample size was 90 (**Figure 1C**).

### 3.3 Diversity of gut microbiota in patients with TB and healthy controls

**Figure 2:**
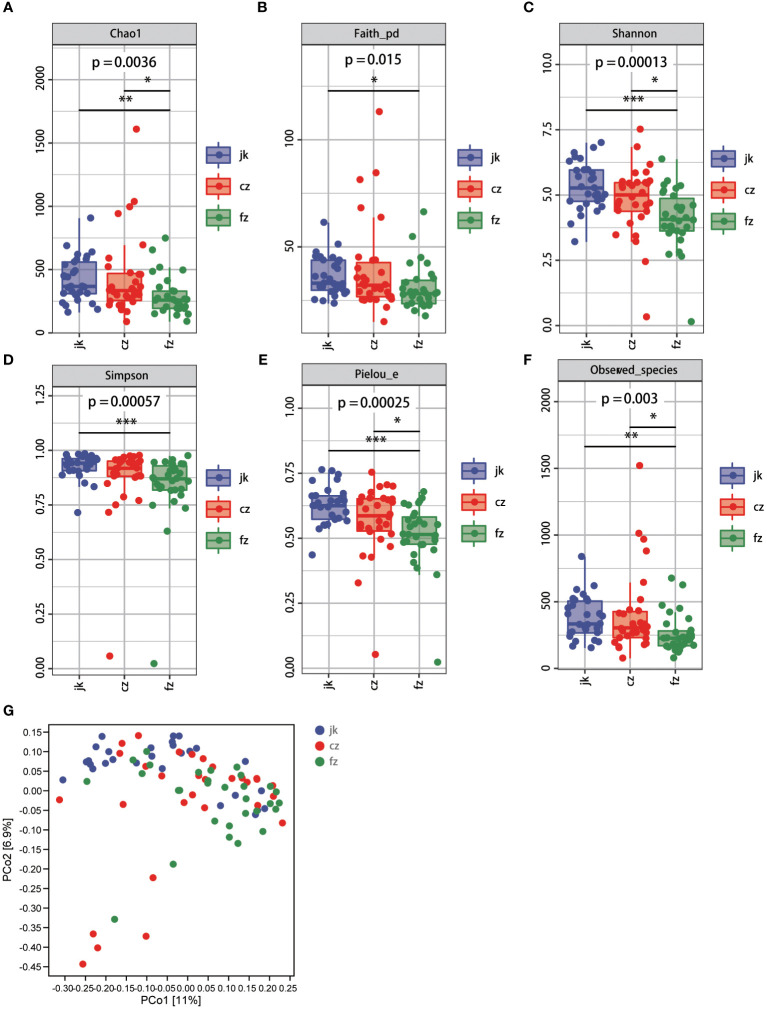
Changes in bacterial diversities in gut microbiota in the patients in different periods of tuberculosis compared with healthy controls. **(A)** Comparison of Alpha-diversity (as assessed by the Chao1 index). p = 0.0036. Wilcoxon rank-sum test. **(B)** Comparison of Alpha-diversity (as assessed by the Faith_pd index). p = 0.015. Wilcoxon rank-sum test. **(C)** Comparison of Alpha-diversity (as assessed by the Shannon index). p = 0.00013. Wilcoxon rank-sum test. **(D)** Comparison of Alpha-diversity (as assessed by the Simpson index). p = 0.00057. Wilcoxon rank-sum test. **(E)** Comparison of Alpha-diversity (as assessed by the Pielou_e index). p = 0.00025. Wilcoxon rank-sum test. **(F)** Comparison of Alpha-diversity (as assessed by the Observed_species index). p = 0.003. Wilcoxon rank-sum test. **(G)** The plots based on UniFrac distances. Green plots represent patients, red plots represent healthy controls. cz, initial TB patient; fz, recurrent TB; jk, healthy control.

Subsequently, we conducted a comprehensive evaluation of the overall diversity using the Chao1-diversity, Faith_ pd- index, Shannon, Simpson, Pielou_ e-index, and Observed_ species indices. The presence of alpha diversity in the gut indicated a notable disparity in the fecal microbiota between patients with different stages of TB and the control group. The healthy control group exhibited a moderate increase in all indices compared to the patients in the recurrent TB group (**Figures 2A–F**, Wilcox test, p < 0.05). The Chao1-diversity, Shannon diversity, Pielou_e-index, and Observed_species-index were significantly increased in the different periods of TB groups (**Figures 2A, C, E, F**, Wilcox test, p < 0.01).

According to the unweighted PCoA (UniFracPCoA), there was no substantial variation observed in the fecal microbial communities between the two groups. The results of **Figure 2G** demonstrate that TB had an impact on the microbiota of the cz and fz groups, as well as a few samples that overlapped.

### 3.4 Gut bacteria as biomarkers in the healthy controls and for the different periods of TB in patients

**Figure 3:**
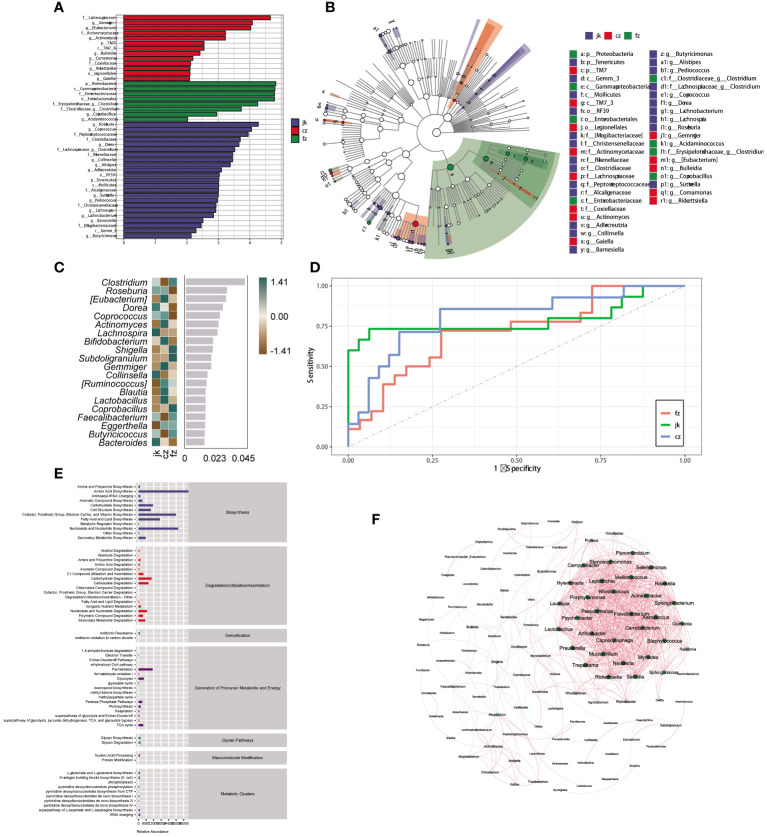
Identification of different genera as biomarkers by relative abundance. **(A)** The column length signifies the impact of distinct species in terms of relative abundance (linear discriminant analysis scores > 4). **(B)** The clado-gram displays the species that are markedly distinct. Each circle symbolizes the phylogenetic progression from phylum to genus, encompassing both the interior and exterior. The size of each circle is linked to the number of taxa present, and the biomarker is in agreement with the group indicated by the color. **(C)** The top 20 bacterial taxa identified using random forest classification of the relative abundance of the gut microbiota in patients and healthy controls. Biomarker taxa are ranked in order of importance to the accuracy of the model based on the mean decrease Gini index. **(D)** The predictive receiver operating characteristic (ROC) curves generated using the 20 candidate biomarkers contributing to tuberculosis progress. Area under the ROC Curve (AUC) (cz, 80.74%) >AUC (jk, 78.96%) > AUC (fz, 71.74%). **(E)** Kyoto Encyclopedia of Genes and Genomes pathway of the enrichment. **(F)** The interactions of the microorganisms. The line between nodes shows positive correlations (red lines) or negative correlations (green lines). Each dot is a different color, representing a different genus of bacteria. The size of the node is directly linked to the average relative abundance of the genus within the enriched population.

To identify the key biomarkers that were differentially expressed among the three groups, we used LfSe to distinguish between the various species in the groups (**Figures 3A, B**). The three groups showed significant differences in 44 taxa. The initial TB patient group showed a unique microbiota, with a higher abundance of Lachnospiraceae, *Gemmiger*, *Eubacterium*, Actinomycetaceae, *Actinomyces*, TM7, TM7_3, *Bulleidia*, *Comamonas*, Coxiellaceae, *Rickettsiella*, Legionellales, and *Gaiella*. Proteobacteria, Gammaproteobacteria, Enterobacteriaceae, Enterobacteriales, *Clostridium*, and *Acidaminococcus* were mainly concentrated in the intestines of retreated patients. After analyzing the relative abundance, it was determined that 20 genera were present, with 12 of them showing an increase in the number of patients in the TB group over different time frames (**Figure 3C**). Twenty genera were used as microbiome markers to distinguish the patients in different periods of TB from healthy controls. We accurately differentiated patients from healthy controls using this model, the area beneath the ROC curve was measured to be 0.807 and 0.715, respectively (**Figure 3D**).

### 3.5 Intestinal microbial function and interaction

Patients with TB exhibited differential enrichment in 61 metabolic pathways compared to healthy controls, with notable disparities (p < 0.05) observed among the three groups (**Figure 3E**). The seven metabolic categories that these pathways fell into were biosynthesis, degradation/utilization/assimilation, detoxification, generation of precursor metabolites and energy, glycan pathways, macromolecule modification, and metabolic clusters. A SparCC network plot was generated to investigate the possible connections between differentially abundant microorganisms through the correlation of co-abundance and co-exclusion (**Figure 3F**). A correlation network analysis was conducted using bacteria with significant differences in the intestinal microorganisms. The majority of the correlations among the bacteria were positive. The evidence points to a complex relationship between these microorganisms; however, further investigation is necessary to gain a more comprehensive comprehension of these associations.

### 3.6 Correlation between microbiota, cytokine, and lymphocyte subsets

**Figure 4:**
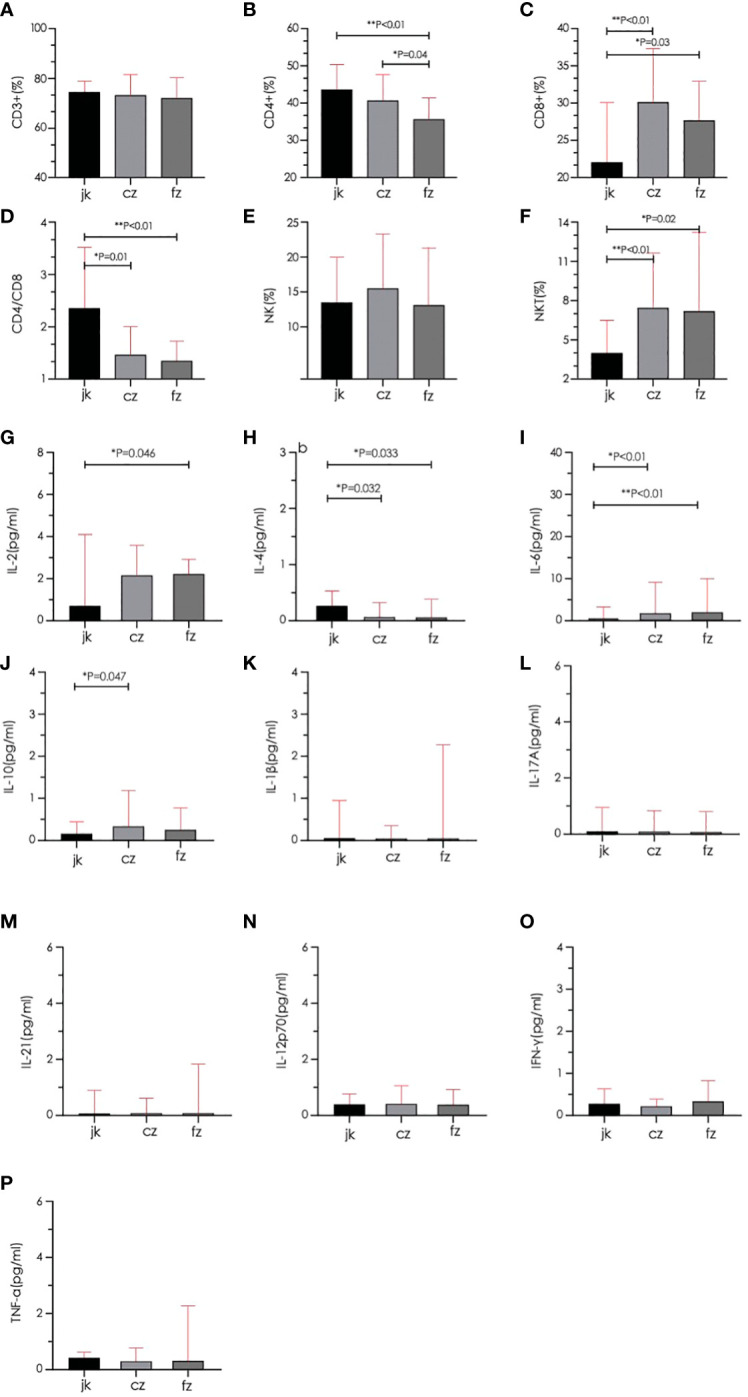
Comparison of lymphocyte subsets and cytokines between different groups. **(A–D)** Total T cell, CD4+ T cell, CD8+ T cell, and CD4/CD8 were quantified in the three groups. **(E, F)** Natural killer cell (NK) and Natural killer T cell (NKT) were quantified in the three groups. **(G–P)** Serum cytokines (IL-2, IL-4, IL-6, IL-10, IL-1β, IL-17A, IL-21, IL-12p70, IFN-γ, TNF-α) were quantified in the three groups. cz, initial TB patient; fz, recurrent TB; jk, healthy control.

**Figure 5:**
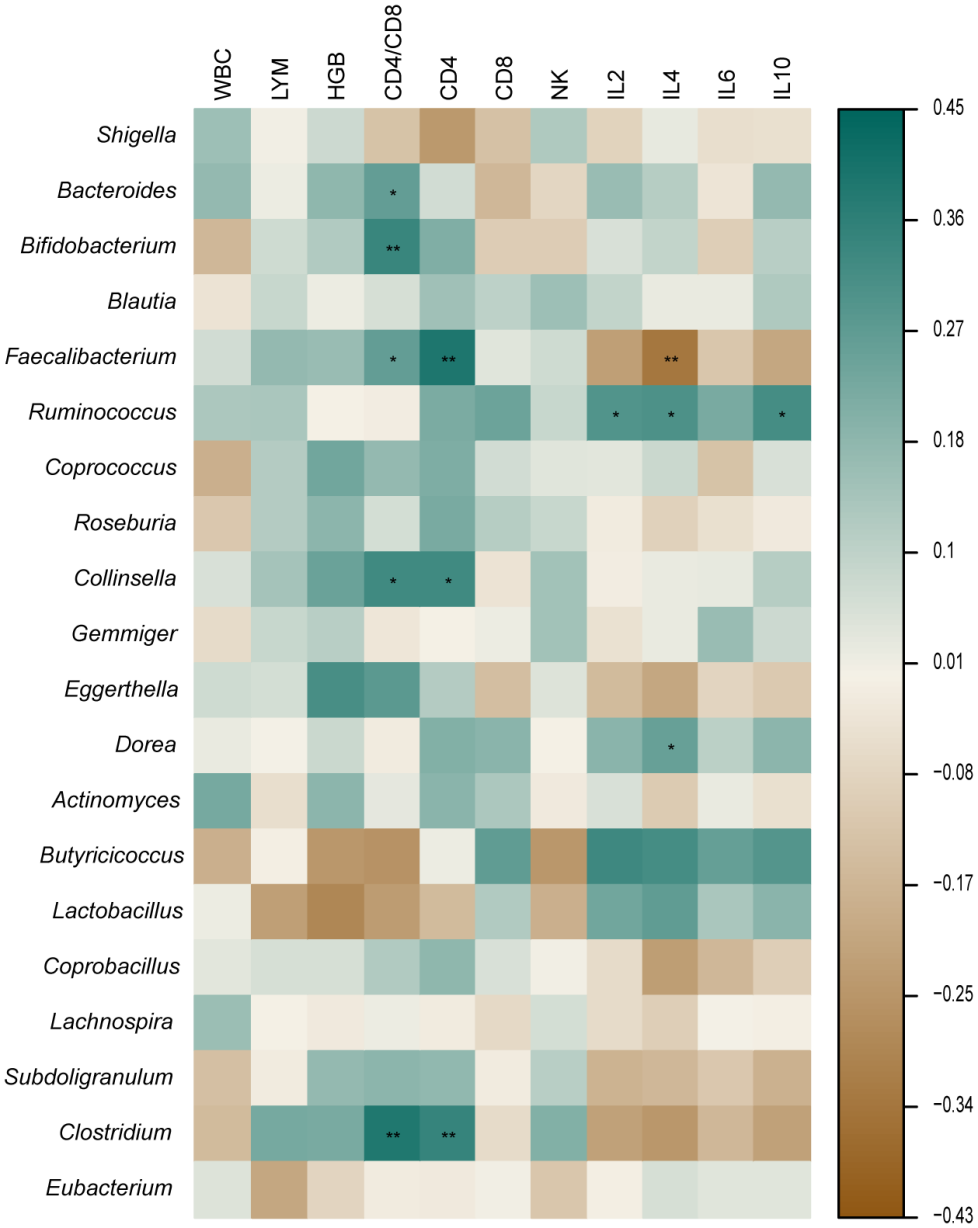
Correlation between microbiota, cytokine, and lymphocyte subsets. *p < 0.05 **p < 0.01.

The retreatment group showed a marked reduction in the amount of CD4+ cells when compared to both the initial treatment and control groups (p < 0.05). The two TB groups had a greater proportion of CD8+ and NKT cells than the control group (p < 0.05), while the CD4/CD8 ratio was substantially inferior to that of the control group (p < 0.05). The cytokine results showed that the control group had a significantly higher IL-2 level than the retreatment group, and the IL-10 level was substantially inferior to the initial treatment group. The IL-4 level in the two TB groups exhibited a significant decrease compared to the control group (p < 0.05), while the IL-6 level displayed a significant increase compared to the control group (p < 0.05). The other indicators showed no noteworthy distinctions (P > 0.05) (**Figure 4**). The analysis of cytokine and lymphocyte subsets is essential for uncovering the possible interaction between the gut microbiota and the host. The results of Spearman’s correlation analysis between cytokines, lymphocyte subsets, and microbiota, which showed significant differences, are shown in **Figure 5**. Five bacterial taxa (*Bacteroides*, *Bifidobacterium*, *Faecalibacterium*, *Collinsella*, and *Clostridium*) were most closely associated with CD4/CD8, while three bacterial taxa (*Faecalibacterium*, *Collinsella*, and *Clostridium* were most closely associated with CD4. Three bacterial taxa (*Faecalibacterium*, *Ruminococcus*, and *Dorea*) were most closely related to IL4. *Ruminococcus* was closely associated with IL2, IL4, and IL10.

## 4 Discussion

Tuberculosis is a serious infectious disease that is endangering human health (23). China has a high burden of TB, with morbidity and mortality rates among the highest worldwide. At present, the BCG vaccine, which is extensively used worldwide, has a certain effect on the prevention of TB in children; however, its effect on adult TB is limited, which is an important reason for the difficulty in effectively controlling the TB epidemic. Therefore, there is an urgent need to explore new methods for the prevention and treatment of TB.

An imbalance of microbes in the intestine can lead to a variety of diseases, such as cancer, cardiovascular disease, obesity, and non-alcoholic fatty liver disease (24, 25). Moderate intestinal flora imbalance is often accompanied by a downregulation of short-chain fatty acid-producing bacteria and an increase in opportunistic pathogens in patients with diabetes (26). Disordered intestinal microecology can increase the incidence of autoimmune diseases and accelerate their progression (27). Disordered intestinal microecology plays an accelerated role in the pathogenesis of hepatitis B (28, 29).

A balanced intestinal microecology can strengthen the body’s resistance to acute and chronic respiratory infections caused by pathogenic bacteria (30), such as asthma, TB, chronic obstructive pulmonary disease, and other chronic lung diseases. Studies have confirmed that mice with intestinal microbial interference are more likely to be infected with MTB, which affects their immune function (31). The connection between intestinal microecology and lung diseases has been referred to as the “gut-lung axis. Nevertheless, the precise correlation between intestinal flora and TB remains ambiguous, and numerous fundamental scientific inquiries persist concerning the practical implementation of microbial therapy in TB treatment.

Gut microbes influence both local and systemic immunity (32). The gut microbiome regulates, optimizes, and suppresses host immune responses, and these changes allow infectious diseases to gain the upper hand (33, 34). Recent studies have linked an increase in intestinal microbiota *Prevotella* at mucosal sites to local and systemic inflammation (35–38). The genus *Prevotella* is a Gram-negative anaerobe, one of the three dominant bacteria in the gut (39), and the main genus in the respiratory system (40). It was discovered in research on respiratory system diseases that *Prevotella* is extensively found in the lower respiratory tract (41). Research has demonstrated that a heightened presence of *Prevotella* is linked to inflammatory disorders (42) is connected to Th17 cell-induced mucosal inflammation, and can cause an upsurge in Th17 cells and IL-17A production in the colon of mice (43). In a study on lung diseases, *Prevotella* was largely responsible for controlling Th17 cells and cytokines. When *Prevotella* is present in abundance, TLR2 is mainly triggered to secrete cytokines, such as IL-1β, IL-6, and IL-23, through antigen-presenting cells, thus prompting CD4+T to differentiate into Th17 cells (44). *Prevotella* also stimulates IL-6 and IL-8 production in epithelial cells and promotes mucosal Th17 immune responses and neutrophil aggregation. Recent studies have shown that the concentrations of propionate and butyrate in the intestine and blood are upregulated in a mouse model of lung cancer, and increased concentrations of propionate and butyrate consequently stimulate the production of chemokine ligand 20 (CCL20) in lung endothelial cells. Subsequently, CCL20 stimulates the influx of Th17 cells into the lungs via receptor 6 (45). *In vitro* experimental studies confirmed that *Prevotella* can stimulate bone marrow-derived dendritic cells to secrete cytokines, causing primitive Th cells to produce numerous Th17 cells (46).

The development and outcome of a human becoming infected with MTB are not only closely related to the degree of MTB virulence but also to their level of immunity (47). Protective immunity against TB is mainly composed of T lymphocyte-mediated cellular immunity and B cell-mediated humoral immunity. CD4+ T cells play a crucial role in cellular immunity as the primary effector of T cells (48). After activation, CD4+ T cells secrete a range of cytokines to regulate MTB, and the amount of these cytokines can significantly influence the course of the illness. Some scholars have observed that a decrease in CD4+ cells may activate latent MTB (49), and the weakening of T cell signals exposed to active antigens for an extended time leads to “immune fatigue” (50), accelerating the deterioration of the disease in both directions. CD8+ cells are cytotoxic T cells that produce granin and perforin, which kill pathogenic bacteria. NKT cells have surface markers for both T and NK cells and connect the link between innate and adaptive immunity (51). The results of our study showed that the amount of CD4+ T cells in the retreatment TB group was substantially inferior to that of the initial treatment and control groups. The two TB groups had significantly higher proportions of CD8+ T cells and NKT cells compared to the healthy control group. Peripheral blood CD8+ percentage and NKT cells may be indicators for the clinical auxiliary diagnosis of active pulmonary TB (52), whereas CD4+ percentage decreases with the aggravation of the disease, which can be used as an indicator to judge the severity of pulmonary TB. In addition, The findings of this research indicated that the CD4/CD8 ratio between the two TB groups was substantially inferior to that of the control. Because of the different immune functions of CD4+ and CD8+, the CD4/CD8 ratio is used to evaluate the immune level of the body, which represents the balance between the purpose of helper T cells and suppressor T cells. The disruption of this balance can lead to immune dysfunction. Decreased resistance to MTB leads to the beginning of illness. The findings indicated that patients with pulmonary TB had immune dysfunction, particularly in the retreatment group. In the future, to gain a better comprehension of the link between T cell subsets and pulmonary TB recurrence, it is essential to enhance the tracking of T cells in individuals with primary pulmonary TB.

In addition, T lymphocyte subsets secrete different cytokines to form a balanced cytokine network (53), and Biomarkers can be utilized to differentiate between various phases of the illness by analyzing cytokine profiles. The findings indicated that the amount of IL-4 in the two TB cohorts was notably reduced, and the amount of IL-6 was notably greater than that of the control group. The initial treatment group experienced a substantial rise in IL-10 levels, while the retreatment group witnessed a significant increase in IL-2 levels. However, the immune role of IL-4 in pulmonary TB remains controversial. The creation of IL-4 by Th2 cells primarily facilitates the differentiation of Th0 cells into Th2 cells. On the one hand, it positively regulates the anti-TB response; on the other hand, as an anti-inflammatory factor, IL-4 can induce macrophages to accelerate apoptosis when the disease is severe, leading to pathogenic damage. Our results favor the latter, with IL-4 selectively controlling antigen presentation to inhibit Th1 cell function, maintain Th2 cell proliferation, cause immune damage, and even form cavitary lesions of TB. IL-6 is induced and maintained as a pro-inflammatory factor during the inflammatory response in TB (54). Numerous studies have demonstrated a direct association between the severity of TB and IL-6 levels. Our research showed that the retreatment group had a considerable rise in IL-6 concentrations When juxtaposed with the initial treatment and control groups, suggesting that the development and outcome of pulmonary TB may be related to its overexpression. IL-2 promotes cytotoxic T cells and their killing activity. Studies at home and abroad have shown that IL-2 in active pulmonary TB is substantially inferior to that in healthy individuals, and IL-2 is low in both new and old pulmonary TB (55). This is consistent with our results, considering insufficient IL-2 secretion, the body was unable to completely clear MTB from evolving into active TB or relapsing. The IL-10 concentrations in the initial treatment group were remarkably superior to those in the control group. The increase in IL-10 in the early stages of TB inhibited the rapid replication of MTB and played a protective role against TB.

This study had some limitations. We did not use the distinguished gut microbes to validate experiments in mice. This study did not analyze the lung microbiota; thus, more research is required to clarify whether the lung microbiota contributes to TB and whether the gut and lung microbiota influence the degree of lung injury in patients with TB.

## 5 Conclusion

In conclusion, there are differences in the microorganisms, T cell subsets, and cytokines, with different relative abundances and structural compositions, between healthy controls and patients during different periods of TB. Gaining insight into the function of the gut microbiome, T cell subsets, and cytokines may help modulate therapeutic strategies for TB.

## Data availability statement

The datasets presented in this study can be found in online repositories. The names of the repository/repositories and accession number(s) can be found below: https://www.ncbi.nlm.nih.gov/, PRJNA1027111.

## Ethics statement

Ethical approval was not required for the studies involving humans because All specimens in this study were from clinical studies. The studies were conducted in accordance with the local legislation and institutional requirements. The human samples used in this study were acquired from primarily isolated as part of your previous study for which ethical approval was obtained. Written informed consent to participate in this study was not required from the participants or the participants’ legal guardians/next of kin in accordance with the national legislation and the institutional requirements.

## Author contributions

YC: Methodology, Writing – original draft, Writing – review & editing. XL: Resources, Writing – review & editing. GB: Investigation, Writing – review & editing. NZ: Resources, Writing – review & editing. DL: Software, Writing – review & editing. XZ: Resources, Writing – review & editing. ML: Software, Writing – review & editing. KL: Data curation, Writing – review & editing. HL: Project administration, Writing – review & editing.
